# Ultrasound combined with microwave irradiation: Cavitation regimes and acoustic emissions^☆^

**DOI:** 10.1016/j.ultsonch.2025.107566

**Published:** 2025-09-17

**Authors:** Dwayne Savio Stephens, Adriano Troia, Giancarlo Cravotto, Katia Martina, Robert Mettin

**Affiliations:** a Drittes Physikalisches Institut, Georg-August-Universität, Friedrich-Hund-Platz 1, 37077 Göttingen, Germany; b Ultrasounds & Chemistry Lab. Advanced Materials and Life Science, I.N.Ri.M., str delle Cacce 91, 10135 Turin, Italy; c Dipartimento di Scienza e Tecnologia del Farmaco and NIS - Centre for Nanostructured Interfaces and Surfaces, University of Turin, Via P. Giuria, 9, 10125 Turin, Italy

**Keywords:** Cavitation, Microwaves, Degassing, Sound emission, Acoustic spectrum, High-speed observations

## Abstract

The irradiation of water by intense ultrasound (US) without and with microwave (MW) heating is investigated by analysis of acoustic emission spectra and high-speed imaging of cavitation bubbles. To this end, pure airborne sound detection proves sufficient for a rough assessment of cavitation quality generated by a 20.5 kHz glass horn inside a MW oven. Results show essentially two cavitation states: soft (or gassy) and hard (inertial) cavitation. Application of US alone leads to soft cavitation in strongly pre-heated water, and to hard cavitation otherwise. The addition of MW irradiation to hard cavitation triggers after a certain delay time the transition to soft cavitation, but a return to hard cavitation is observed after switching MW off. The findings are discussed in the context of water temperature and relative air saturation of the liquid. It is conjectured that rapid MW heating during US irradiation can drive the water into stronger oversaturation, while US alone does not. Further experiments for exploration of the observed effects are suggested, and potential optimization strategies for US/MW applications are proposed.

## Highlights

• Demonstration of feasibility of acoustic cavitation diagnosis in the 20 kHz range via airborne sound.
• Acoustic and visual analysis of ultrasonic cavitation combined with microwave irradiation.
• Switching microwave irradiation on and off can transfer inertial cavitation to gassy cavitation, and back.

## Graphical abstract

**Figure:**
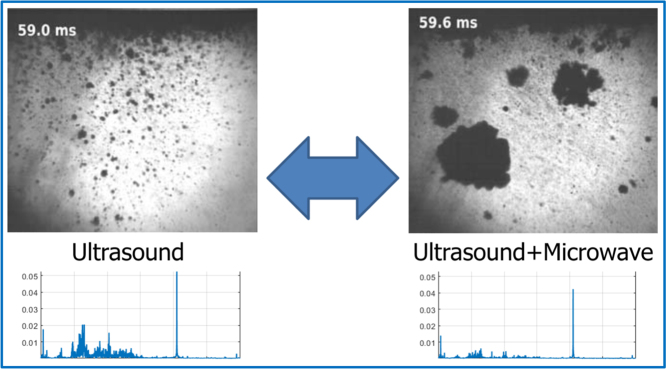

## 1 Introduction

Ultrasound (US) irradiation and microwave (MW) heating of aqueous solutions have shown synergetic effects and can promote advanced processing when applied simultaneously or sequentially [1].

Chemists have long sought new forms of synergy by combining tools and processes to improve efficiency, selectivity, safety, economy and sustainability. The integration of ultrasound (US) and microwave (MW) energy is based on their complementary properties: MW enables rapid heating but is limited by mass transfer, while US enhances mass transfer but provides minimal heating. By combining the two in a single system, these limitations can be overcome [1].

The first evidence of the synergistic effects of simultaneous US/MW irradiation was reported in 1995 by Maeda and Amemiya [2] who monitored its effect through observations of sonoluminescence and chemiluminescence. Around the same time, Jacques Berlan et al. were also conducting pioneering research on combining these two techniques [3]. In both cases there was a significant improvement in organic synthesis when reactions were performed under US/MW promotion. The two techniques were demonstrated to complement each other: acoustic cavitation from US generates concentrated energy through localized hotspots, while MW enables selective dielectric heating.

Attempts to combine US/MW irradiation have involved the application of both energies sequentially, with the reaction mixture circulating through separate compartments in loop or flow mode, or simultaneously, with a single reactor in which the reaction medium is irradiated by US within the MW cavity [1], [4], [5]. The simultaneous approach is considered to be the most effective for maximizing efficiency [5].

The use of hybrid reactors integrating both irradiation techniques has therefore gained increasing attention, particularly in extraction [6], organic synthesis [7], [8], [9], biodiesel production [10], [11], [12] and biomass conversion [13]. In addition, applications in food processing [14] and nanoparticle synthesis have expanded significantly over the last decade [15], [16].

Recent publications evidence the widespread use of water as the preferred solvent for combined MW/US extraction [17], [18], food processing [19], [20] and nanoparticle preparation [21]. A thorough understanding of the interactions between MW and US irradiation with the solvent is crucial for optimizing and scaling up these processes. Understanding the key parameters that enhance the synergistic effects is essential for improving the efficiency of this approach. To the best of our knowledge, only one study has addressed this problem by a thorough simulation of heat deposition by US and MW absorption and liquid flow due to convection and acoustic streaming [22]. The authors report a significant enhancement of temperature homogeneity in the reactor by combined MW and US application via acoustic streaming, as compared to MW application alone. The detailed role of cavitation, however, is not yet well investigated, possibly also because the access to the MW system is rather limited.

In the present work, we try to characterize in more detail the acoustic cavitation phenomena that occur in combined US/MW setups. Our aims comprise a better understanding of the physical effects and also a suitable way to monitor the cavitation activity of the system. To this end, we conducted several types of experiments in which water samples were sonicated by a Pyrex glass sonotrode horn emitter, with and without MW irradiation. The results show a clear effect of liquid temperature and respective relative air saturation on the cavitation type, namely gassy vs. inertial cavitation. As a rather unexpected feature, we observed a reversible switching from inertial to gassy cavitation during MW application. This finding might indicate that microscopic effects of MW bulk heating are not yet fully understood, particularly the effects on dissolved gas. Since the observed transition of cavitation regimes happened only after certain temporal delays, an improvement of existing US/MW processes might be possible by modification of the driving schemes, e.g. towards shorter MW application intervals.

**Fig. 4:**
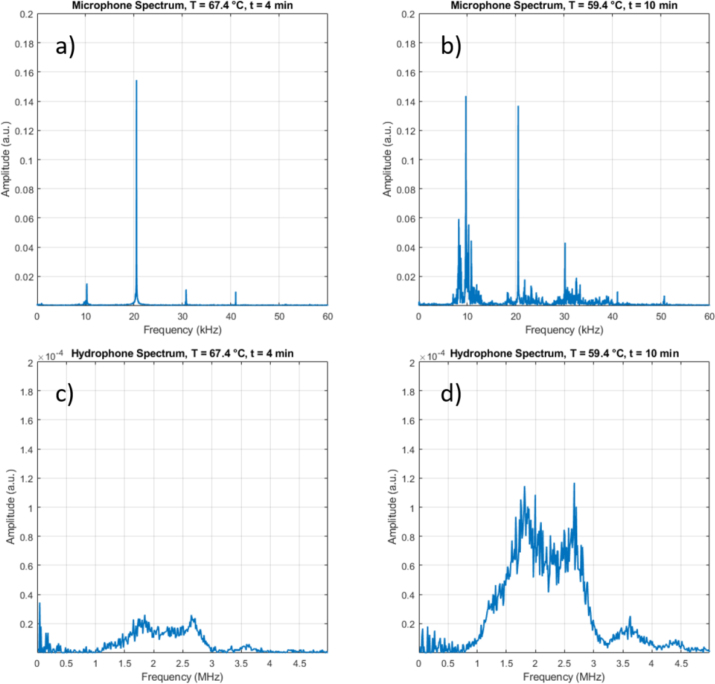
Illustration of acoustic spectra for gassy vs. inertial cavitation, taken from the experiment discussed below in Fig. 6. Left frames: (a) air microphone and (c) hydrophone spectra for gassy cavitation (recorded after 4 min of the run, at T=67.4°C). Right frames: (b) air microphone and (d) hydrophone spectra for inertial cavitation (recorded after 10 min of the run, at T=59.4°C).

## 2 Experimental methods

**Fig. 1:**
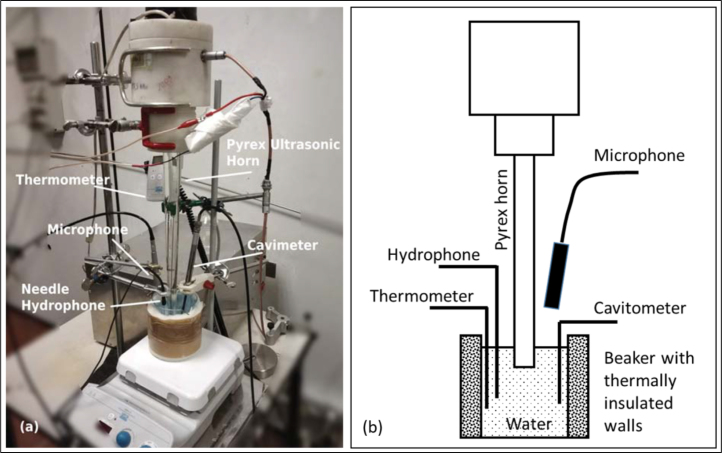
Experimental setup I as photograph (a) and schematic (b). A cylindrical beaker with non-transparent wall insulation is accessed from top with the glass horn. The setup allowed for insertion of a thermocouple, a needle microphone, and the sensor of a cavitometer. The air microphone was positioned closely above the water surface. High-speed recordings of cavitation were not possible.

**Fig. 2:**
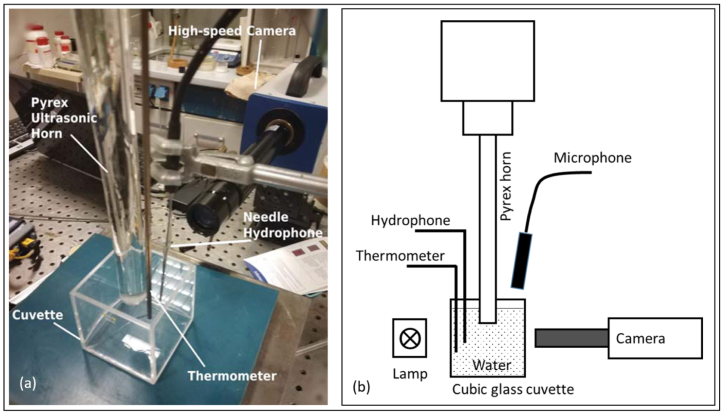
Experimental setup II as photograph (a) and schematic (b). A cubical glass cuvette allows for insertion of thermocouple, needle microphone, and high-speed recordings from the side. The air microphone is not shown in (a).

**Fig. 3:**
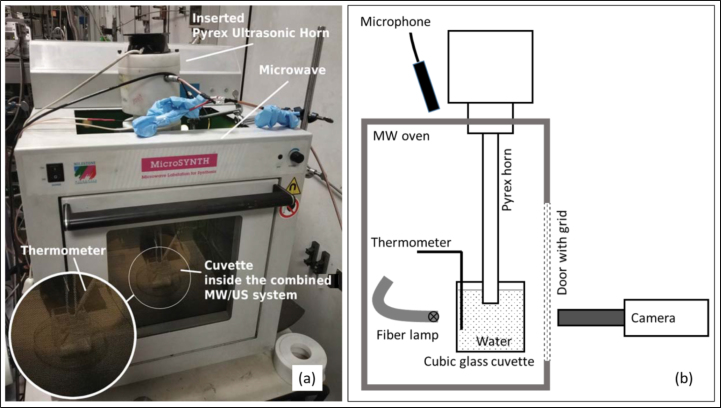
Experimental setup III: Photograph (a) and schematic (b) of the custom MW oven device with the Pyrex horn inserted from top. The cuvette (see magnified inset) was placed inside the combined MW/US system with a special fiber optical thermometer that was used to record the water temperature over time. A fiber optic light source was used to back-light the cuvette. The camera was placed just outside the glass window. Due to the protective mesh behind the glass window, the field of view that the camera could record was hampered.

Experiments were conducted at the Acoustics and Ultrasound Lab at Istituto Nazionale di Ricerca Metrologica Italy (INRIM), Turin, and in the Dipartimento di Scienza e Tecnologia del Farmaco and NIS, University of Turin. To cope with the restrictions of a MW oven, inside of which metal parts are not admissible, three different setups (I, II and III) were investigated that allowed for different preparations and accessibility by measurement devices, see Fig. 1, Fig. 2, Fig. 3. The acoustic driver was always a specially manufactured Pyrex glass horn transducer (Danacamerini s.a.s., Italy) with 18 mm tip diameter. The horn was dipped vertically from top into the open receptacles of the liquid, with the tip positioned 1 cm below the free surface. The device was running at its resonance at 20.5 kHz with an adjustable voltage. The resulting transmitted electrical power could be read off the amplifier and could range between 1 and 50 W. In the combined US/MW experiments, the voltage was fixed, but the power delivered by the transducer was observed to slowly change in time. This was apparently caused by thermal and acoustic impedance drifts. The time traces of the US power had been logged then and are shown in the according figures.

Acoustic recordings were done in water with a needle hydrophone (Dapco NP 10-3) if access was possible, and in air with a microphone Bruel & Kjaer B & K 4166 with B & K 2669 pre-amplifier, connected with a B & K 5935 amplifier. The signal of the hydrophone was sent to an Agilent N9320B spectrum analyzer, and the microphone to an Agilent DSO-X-2022 A oscilloscope. The needle hydrophone traced the acoustic emission in water above 500 kHz and up to 5 MHz, while the microphone was used in air for 0 to 80 kHz. The microphone bandwidth thus included the audible frequency range as well as an ultrasonic range.

**Fig. 5:**
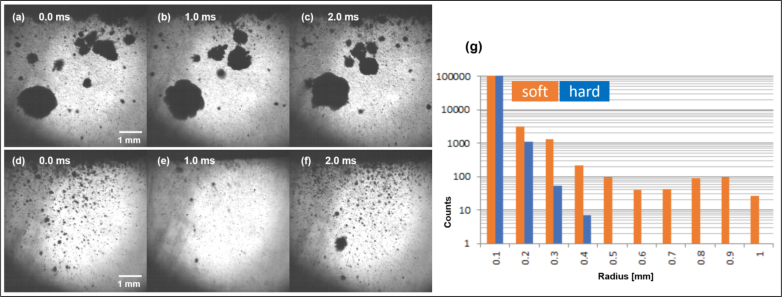
Left: Soft cavitation and hard cavitation in an experiment in setup II, similar to the run presented in Fig. 6, but US power 45 W. Top movie frames (a), (b), (c): soft cavitation; bottom movie frames (d), (e), (f): hard cavitation (times and scales indicated). The disappearance of many small bubbles between frames (d) and (e) highlights their oscillation with a strong collapse, a feature not observed for the larger bubbles in (a), (b). Longer sequences are given in Appendix B, and both movies are provided as Supplementary Material. (g): Bubble radius statistics evaluated over 2000 subsequent frames from the same high-speed movies. Orange (left) histogram bars correspond to the soft, blue (right) bars to the hard cavitation state. Every frame was evaluated, and thus bubbles at different expansion or collapse state are included. Still, a clear difference is obvious. Bubble radii larger than about 0.5 mm were not observed in the hard regime, but were frequent in the soft regime.

In setup I, a cavitation meter (cavitometer ICA-5D, BSUIR) was used to measure the “cavitation activity” determined by its own software. The device is essentially a hydrophone that processes the emission spectra and outputs a voltage that quantifies the level of cavitation.

**Fig. 6:**
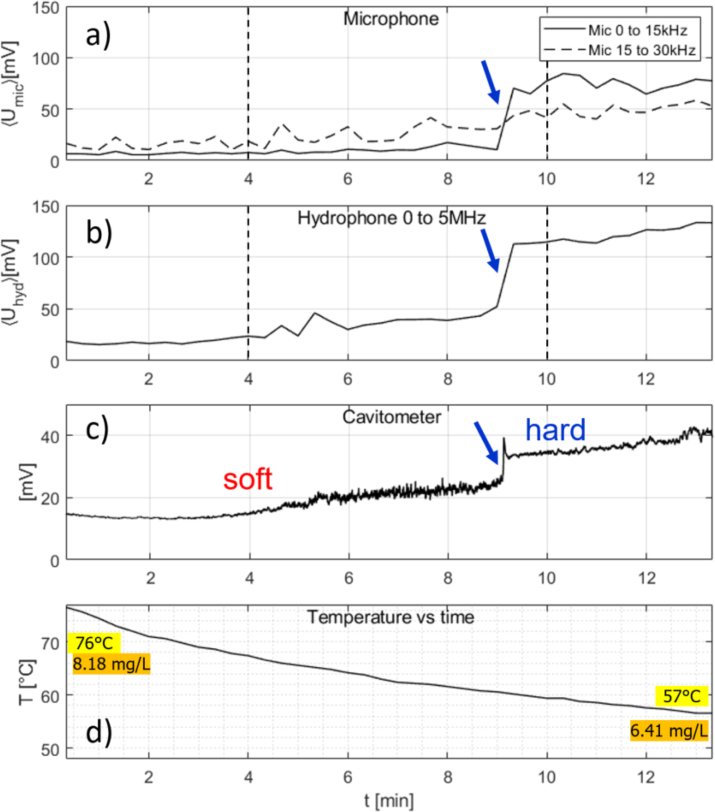
Measurement time traces for pre-heated water (net cooling) under continuous 30 W US application alone (no MW). (a) Air microphone integrated spectrum from 0 to 15 kHz (continuous line) and 15 to 30 kHz (broken line); (b) hydrophone integrated spectrum from 0 to 5 MHz; (c) cavitometer readout; (d) temperature readout. Initial and final temperatures and oxygen concentrations are indicated. Blue arrows mark the transition from soft to hard cavitation. The black dashed vertical lines in (a) and (b) indicate the time instances when the spectra in Fig. 4 had been recorded.

**Fig. 7:**
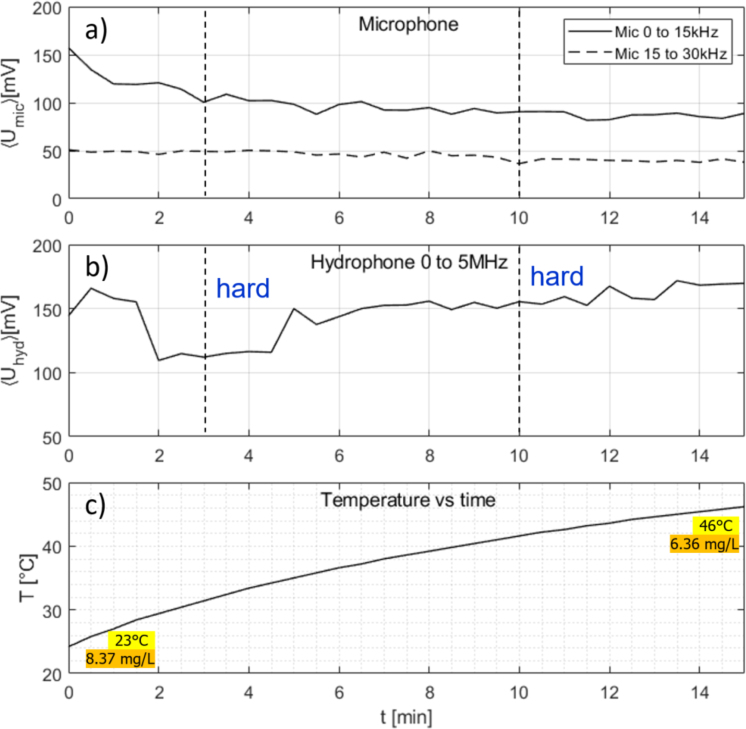
Measurement time traces for room temperature water (net heating) under continuous 30 W US application alone (no MW). (a) Air microphone integrated spectrum from 0 to 15 kHz (continuous line) and 15 to 30 kHz (broken line); (b) hydrophone integrated spectrum from 0 to 5 MHz; (c) temperature readout. Initial and final temperatures and oxygen concentrations are indicated. The cavitometer was not available for this run, but high levels of microphone and hydrophone report hard cavitation throughout. Black dashed vertical lines indicate the time instances where sample spectra are shown in Appendix A, Fig. A.15.

In setups I and II, temperature was accessible during the runs via a thermocouple from a heating magnetic stirrer plate (AREC.X; the heating plate was always turned off). Setup III employed a fiber optic temperature sensor that was a built-in part of the oven. Additionally an OTG-F (OpSens Solutions) was available for checks..

For optical measurements of bubble dynamics and populations, a high-speed camera (Optronics Camrecord 5000) was employed in setups II and III with a long distance microscope (Infinity KC-IF 3) and a macro objective (LINOS MeVis C). The frame rates were 5 kfps and 1 kfps, and the exposure time could be varied between 20μs and 1 ms.

The receptacle in setup I was a cylindrical beaker with thermally insulated, non-transparent walls, see Fig. 1, where high-speed recordings of cavitation were not possible. Setup II used a glass cuvette instead, with clear access for imaging under background lighting. The same glass cuvette was used in setup III inside a modified MW oven. The oven is a custom device, developed in the laboratory of the University of Turin (Dipartimento di Scienza e Tecnologia del Farmaco), where the Pyrex ultrasonic horn is integrated from top into a Microsynth (Milestone s.r.l) microwave chamber [23]. The MW oven had limited visual access from outside via the shielding metal grid, but rough visual information on bubble dynamics could nevertheless be obtained using a bundle of optical fibers inserted into the MW oven and coupled to an LED light source.

The liquid samples were typically 100 ml of filtered DI water. The dissolved air content was determined via oxygen and measured by an oxymeter (Hq40, Hach Lange) before and after most runs. In some of the experiments, the water had been pre-heated in advance by a standard MW oven for about 30 to 60 s, i.e. rather rapidly. While the energy delivery by US and/or MW application usually led to a net temperature rise of the water during the run, in some pre-heated measurements the initially high temperature was actually falling due to passive cooling by heat conduction to the colder environment. Since the valid temperature range of the oxymeter was limited, the dissolved oxygen values of hotter than 40 °C water samples were obtained either before rapid heating, or after letting the sample cool down sufficiently.

## 3 Gassy and inertial cavitation

In the course of the experiments, the most striking effect was the occurrence and alteration between two quite clearly distinguishable states of cavitation: gassy and inertial cavitation. It is well known since long that acoustic cavitation in liquids with higher gas content has a different character than in degassed liquid, see for instance [24], [25]. This difference shows up in various observables: In gassy liquid, many larger bubbles occur, often with surface deformations and weaker volume oscillations. At about 20 kHz driving, the emitted audible sound appears muffled and lower than in degassed liquid. Also, the mechanical action of the gassy cavitation bubbles is less aggressive. Since surface cleaning or erosion effects are weak or even absent in the gassy cavitation regime, it is as well sometimes referred to as “false”, “pseudo” or “soft” cavitation. The arising larger bubbles are sometimes called “degassing” bubbles since their origin is typically outgassing, and thus these bubbles contain a larger amount of non-condensable gas (often air) and less vapor. In contrast, the cavitation in sufficiently degassed liquid generates smaller, mostly spherical and stronger oscillating bubbles. Bubble collapses are more violent, and the audible cavitation noise appears more harsh or sharp then. Since cavitation bubbles in the partly degassed regime contain more vapor and less non-condensable gas, their collapse is less cushioned by rest gas and results in a stronger compression peak. The frequently emitted acoustic collapse shock waves form a main background of the acoustic cavitation spectrum in the shape of many harmonics and high continuous noise floor. This signature can be used, for instance, to assess the cavitation state by hydrophone measurements, and it is typically the basis of all cavitation meter devices [26], [27]. Since bubble collapses are dominated by the inertia of the inrushing liquid (as contrasted to the counter-pushing non-condensable gas), this cavitation regime is also called “inertial” cavitation. Further terms are “true”, “hard” or “vaporous” cavitation. In earlier works, also the terms “stable” and “transient” cavitation for the different regimes occur, but we suggest to avoid them due to their partly misleading implications on the bubble lifetimes. In the following, we mainly use the expressions soft and hard cavitation for the gassy and inertial regimes. The literature on assessment of the acoustic emission spectra of cavitation is large, and we just refer to a few works and the references therein [28], [29], [30], [31].

In our experiments, the discrimination between soft and hard cavitation was accomplished mainly via the acoustic signals from the air microphone and the hydrophone (if possible), supported partly by the cavitometer. A clear distinction of emission spectra of the two cases is presented in Fig. 4, here recorded from setup I. For the gassy state (Fig. 4a), the microphone registers mainly harmonics and the sub-/ultra-harmonics apart from the driving frequency at 20.5 kHz (see [32] for the nomenclature), and a quite small noise floor. Inertial cavitation (Fig. 4b) generates more intense lines and a broader and higher noise level, notably around the subharmonic [29], [33], [34] and below. Although the microphone type was calibrated only up to 16 kHz, it was well able to include higher frequency features, visible here as noisy bands around 20 kHz and 30 kHz, and as lines up to 50 kHz. The detailed spectral features exhibited a certain variability, however, and at this stage of investigation, only integrated measures of the raw spectra were used for assessment. Essentially, the lower range up to 15 kHz was integrated as the “audible” part, and an integration of the higher range between 15 and 30 kHz served as an “ultrasonic” part of the microphone signal.

The hydrophone spectra, recorded in parallel to the microphone data in the same experiment, are given in Fig. 4c (soft cavitation) and Fig. 4d (hard cavitation). With the used needle hydrophone, essentially the high-frequency range up to 5 MHz could be addressed, and as an emission measure, an integration of the raw spectrum over the full bandwidth was employed. As can be seen in the figures, inertial cavitation raises the high-frequency noise level considerably as compared to the gassy state. This is a common feature of the “true” cavitation indicating strong bubble collapses with shocks. In many applications, like object cleaning, such bubble dynamics is beneficial or even necessary [35]. In “hotspot” sonochemistry, usually it is as well assumed that strong bubble implosions are desired, although the chemical action and pyrolysis in the bubble might depend as well on non-spherical bubble motion and liquid spraying into the bubble [36]. With respect to mass transfer enhancement by streaming and micro turbulence, one might assume as well that hard cavitation is beneficial when compared to its soft counterpart.

Additionally to acoustic emission data, visual inspection by high-speed recordings gave further indication of the different cavitation states, when optically accessible. In Fig. 5a–f, example images from setup II are shown with a rather clear distinction between the larger (soft) bubble population and the smaller (hard) bubbles in the inertial regime. The distinction manifests itself in according bubble size statistics, where a clear shift to small bubbles occurs after the transition to hard cavitation; see the diagram in Fig. 5.

## 4 Results

In the following we report on six exemplary experiments to highlight the connection of liquid temperature, US and MW application, and cavitation conditions in terms of soft and hard regimes. More similar runs have been conducted with essentially identical outcomes, which are not shown here.

### 4.1 Sonication of pre-heated water

The water sample had been pre-heated in a standard microwave oven to 76 °C. Application of US alone (30 W) in setup I initiated soft cavitation with acoustic microphone and hydrophone spectra as shown in Fig. 4a and c. Since the liquid was strongly pre-heated, the sample temperature actually decreased permanently during the full run to reach about 57 °C after 13 min, in spite of the energy deposition by the sonotrode. After about 9 min, a sudden upward jump in the acoustic emissions of the microphone and the hydrophone signals occurs, as depicted by the spectra in Fig. 4b and d. This indicated the rather rapid transition to the inertial cavitation regime. Also the cavitometer readout, that had slightly increased already before, shows a peak and a jump. The simultaneous traces of audible and ultrasonic microphone bands, hydrophone, cavitometer, and temperature readouts are shown in Fig. 6 together with indicators of the transition and the cavitation regimes. Initial and end temperatures and dissolved oxygen levels (8.18 mg/L and finally 6.41 mg/L) are given explicitly as well in the plot. Inspection of the values indicates an initial supersaturation of the hot water, but a roughly equilibrated gas level at the end. This is discussed later in Section 5.

### 4.2 Sonication of room temperature water

The water sample was equilibrated to room temperature (23 °C). Continuous sonication in setup I with 30 W led to a gradual increase of temperature, finally to 46 °C after 15 min. This is shown in Fig. 7. Cavitation indicators here show hard cavitation right from the beginning, and the inertial regime persists until the end, although a slight decrease of the integrated spectra in the audible range occurred. In spite of the rising temperature and the accordingly reducing equilibrium air saturation level, the cavitation noise remained quite high. The oxygen measurement after the run showed that indeed the actually dissolved air had fallen roughly to the new equilibrium value. This means that under these conditions, the degassing apparently had happened sufficiently fast under the perpetuated hard cavitation, and no soft cavitation state had been provoked by oversaturation.

**Fig. 8:**
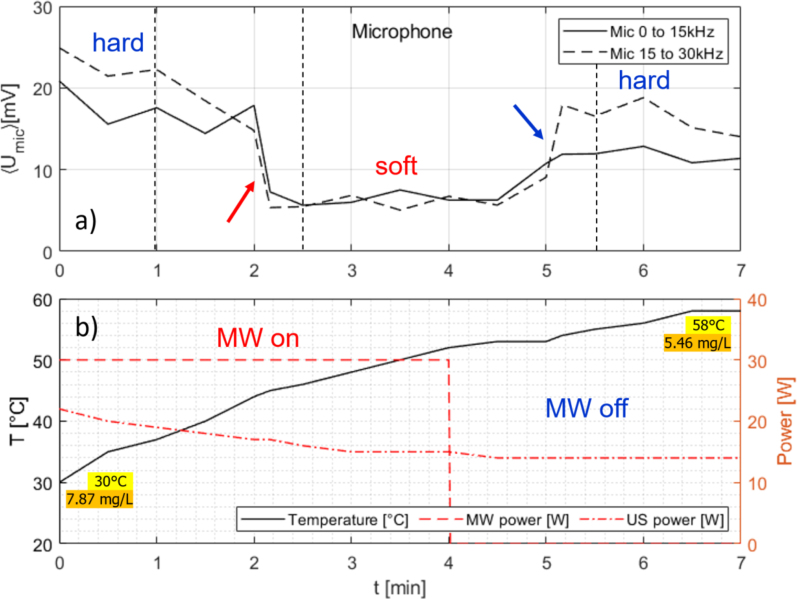
Measurement time traces for room temperature water under continuous US irradiation and additional MW application of 30 W, switched off after 4 min. (a) Air microphone integrated spectrum from 0 to 15 kHz (continuous line) and 15 to 30 kHz (broken line); (b) temperature readout (continuous line, left ordinate) and powers of MW (dashed) and of US horn (dash-dotted). Powers refer to right ordinate. Initial and final temperatures and oxygen concentrations are indicated. The red arrow shows the transition from hard to soft cavitation, the blue arrow the return to hard cavitation. Sample spectra are given in Appendix A, Fig. A.16, for the time instances marked by black dashed vertical lines in (a).

**Fig. 10:**
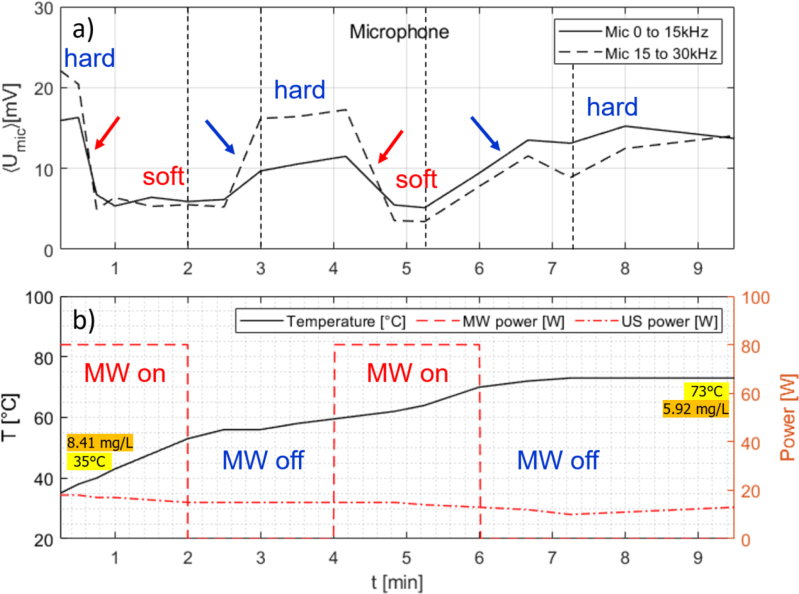
Measurement time traces for slightly pre-heated water under continuous US irradiation and with two times 2 min, 80 W MW on and off switching. (a) Air microphone integrated spectrum from 0 to 15 kHz (continuous line) and 15 to 30 kHz (broken line); (b) temperature readout (continuous line, left ordinate) and powers (right ordinate) of MW (dashed) and US horn (dash-dotted). Initial and final temperature and oxygen concentrations are indicated. The red arrows show the transitions from hard to soft cavitation, the blue arrows the return to hard cavitation. Initial and final temperature and oxygen concentrations are indicated. Black dashed vertical lines in (a) indicate the time instances where sample spectra are shown in the Appendix, Fig. A.17.

### 4.3 Sonication with microwave on–off switching

In setup III, first a constant microwave irradiation of 30 W had been applied during continuous sonication of a room temperature water sample. After 4 min, the MW was turned off, but sonication continued. Only the air microphone could be used as an acoustic sensor, and the spectrally integrated audible and ultrasonic spectra are shown as time traces in Fig. 8 (continuous and broken lines, top graph). Note that in this setup, the absolute acoustic levels are significantly reduced now as compared to Fig. 6, Fig. 7, since the microphone had to be placed outside the MW oven that blocked (apparently about 90%) of the emitted sound. Additionally, Fig. 8 presents the MW and US power and the temperature vs time (bottom graph). With fixed input voltage, the delivered horn power drifted from initially 22 W to finally 14 W. A monotonous rise of temperature from 30 °C at the beginning of the recording to 58 °C after 7 min occurred, with steeper slope under MW irradiation. The outstanding feature of this experiment is the sudden transition from the initial hard cavitation to a soft regime after about 2 min, while MW and US irradiation both continue. The soft cavitation persisted during the full interval of MW application (4 min) and also for a period after MW switch-off. At about the 5 min mark, however, the microphone levels raised, indicating that the inertial cavitation set in again.

**Fig. 9:**
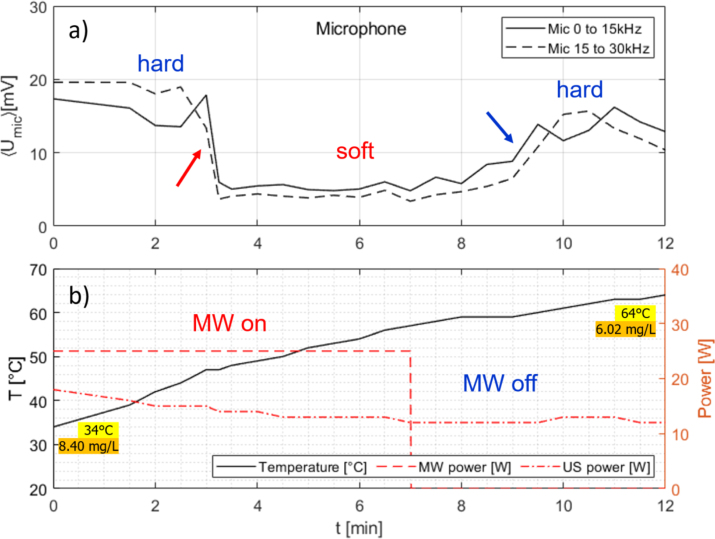
Measurement time traces for room temperature water under continuous US irradiation and additional MW application of 25 W, switched off after 7 min. (a) Air microphone integrated spectrum from 0 to 15 kHz (continuous line) and 15 to 30 kHz (broken line); (b) temperature readout (continuous line, left ordinate) and powers (right ordinate) of MW (dashed) and US horn (dash-dotted). Initial and final temperature and oxygen concentrations are indicated. The red arrow shows the transition from hard to soft cavitation, the blue arrow the return to hard cavitation.

A similar run is presented in Fig. 9, where all parameters differ slightly, but the qualitative behavior remains the same. Here, a 7 min pulse of 25 W MW is applied to weakly pre-heated water, while the sonication runs all the time up to 12 min (with a power between 17 W and 12 W). Again, both transitions from hard to soft and vice versa occur in the experiment.

The dissolved oxygen levels in both runs indicated a slight oversaturation in the beginning and the end, but relatively close to equilibrium saturation, according to the temperatures measured in the run. Therefore, an overall degassing during the temperature rise took place, similar to the experiment in Fig. 7 with US alone. However, during the additional MW episode of heating, soft cavitation emerged (with some temporal delay), whereas it did not appear in the pure US heating case. Furthermore, the soft regime disappeared (again with some delay) under the single action of the sonotrode afterwards. Microwave irradiation and gassy cavitation therefore appear to be coupled somehow in our setup. To further clarify this, a pulsed MW application is probed in the following.

### 4.4 Sonication with pulsed microwave

Again starting with room temperature, the water sample was continuously sonicated in setup III. US power (at constant voltage) ranged from 20 W in the beginning to 13 W at the end. Now the MW was added alternately with higher power (80 W) for periods of 2 min, and then switched off for another 2 min. The pulses were repeated two times in a 9.5 min run. Time traces of acoustic readouts, microwave and US power, and temperature are shown in Fig. 10. The outcome was similar to the single MW pulse (Fig. 8, Fig. 9): After switching MW on and some delay, soft cavitation set in, and hard cavitation returned a short time after ending the MW pulse. Now the delay times of the transitions seem to be shortened and amount to about 30 s rather than 1 min. This might be coupled to the applied MW power, which had been higher in this run, but might be also just a statistical issue. Notably, cavitation returns to the hard regime also after the second pulse, although the water temperature had reached already more than 70 °C. Oxygen measurement indicated 5.92 mg/L after the experiment when the sample had cooled down to 40 °C. In spite of some oversaturation, this was a sufficiently low dissolved gas level to maintain the inertial cavitation (compare the first experiment starting with hot water at 76 °C switching to inertial cavitation at around 60 °C, Fig. 6).

**Fig. 11:**
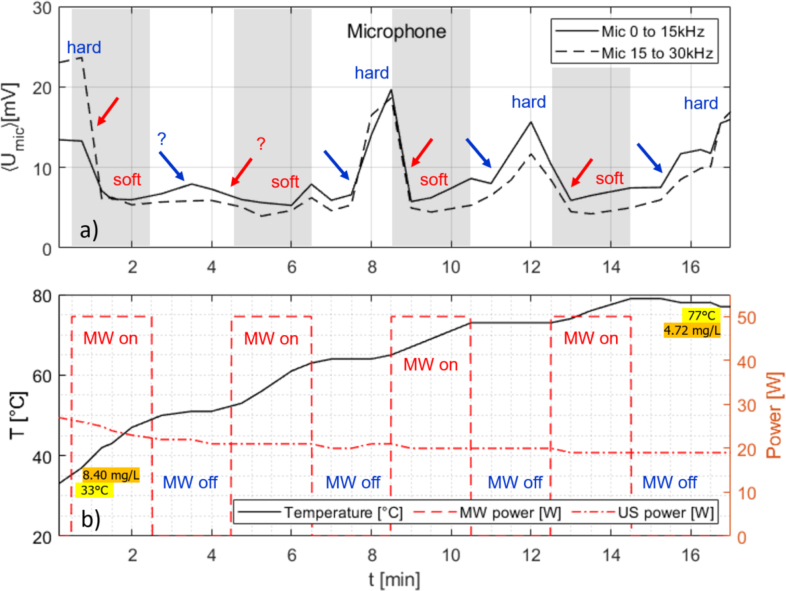
Measurement time traces for slightly pre-heated water under continuous US irradiation and with alternating 2 min, 50 W MW on and off switching. (a) Air microphone integrated spectrum from 0 to 15 kHz (continuous line) and 15 to 30 kHz (broken line); (b) temperature readout (continuous line, left ordinate) and powers (right ordinate) of MW (dashed) and US horn (dash-dotted). Initial and final temperature and oxygen concentrations are indicated. The red arrows show the transitions from hard to soft cavitation, the blue arrows the return to hard cavitation. The acoustic signal change during the first MW off phase is weak, so a switch to hard cavitation and back is doubtful (marked by “?”). In the other intervals, rather clear transitions occurred. For clarity, the MW episodes are shaded in (a).

Another trial with repeated MW pulses under permanent US sonication is shown in Fig. 11. Again a 2 min on–off MW pulsing was done, now over a total runtime of 17 min. The MW power was set to 50 W, and the US power (at constant voltage) drifted downwards from 28 W to 19 W. The temperature was rising from 33 °C to nearly 80 °C during this run, and in the end, the temperature is actually falling when the MW is off. Apart from the first off-interval of the MW, clear transitions show up in the integrated acoustic spectra, marking transitions from hard to soft cavitation and back. The low final oxygen level of 4.72 mg/L lies close to the equilibrium saturation level at 70 °C, and this observation fits to the fact that hard cavitation occurred at such high temperatures. Regime switching delay times after MW on (to soft) and after MW off (to hard cavitation) appear to lie again in the minute range (0.5 to 2 min.).

The possibly “missing” hard cavitation during the first MW off-interval in Fig. 11 might point to a certain statistical behavior, but still an overall systematic can fairly be stated from the experiments reported here, and similar outcomes were found in additional trials (not shown here). The observed variability might stem from inherent fluctuations in the cavitation bubble field, or from further uncontrolled parameters, like water cleanliness or MW hot spots.

### 4.5 High-speed recordings in the MW setup

**Fig. 12:**
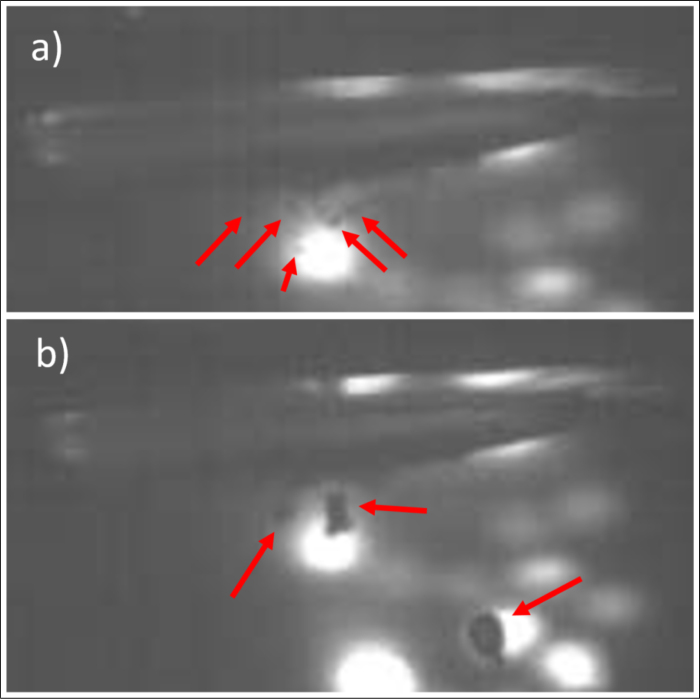
Optical recordings in setup III from within the MW oven for (a) hard and (b) soft cavitation states. Frame width about 20 mm, the horn tip is visible in the top. The arrows mark cavitation bubbles in the low contrast images. Similarities with the recordings from setup II are striking, see Fig. 5.

**Fig. B.20:**
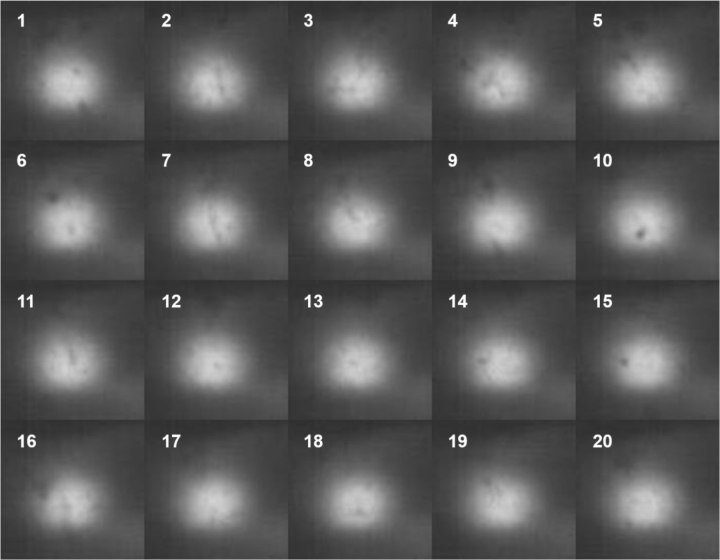
Frames from a high-speed recording series of hard cavitation in setup III. Frame width 4.4 mm, interframe time 1 ms, exposure time 1 ms. ((Link to the movie “HardCavitation_in_MW.avi”)).

The measurements in the closed MW apparatus had to be done without hydrophone or cavitometer access, and thus the information on the cavitation state were relying only on the air coupled sound received with the microphone, that moreover was partly blocked by the closed box. Still, the integrated spectra of audible and ultrasonic ranges represented appraisable quantities of major variation. To verify their significance, also optical recordings were performed in setup III for US (30 W) and simultaneous MW heating with high power (100 W and 200 W) of room temperature water. High-speed movies were taken before and after the clearly measurable transition from high to low acoustic noise floor, i.e. apparently from hard to soft cavitation, provoked by the rapid heating and strong oversaturation. Two movie sequences are contained in the Supplementary Material, and we show some frame sequences in Appendix B. Fig. 12 shows two sample frames, and indeed one can perceive fairly well the same distinction of bubble size and population as in Fig. 5: smaller inertial (hard) bubbles in Fig. 12(a) and larger (soft) degassing bubbles in Fig. 12(b). From the movies, additionally the stronger collapse of the small bubbles can be detected (compare Fig. B.20). Our conclusion is that a significant airborne spectral discrimination of the indicated cavitation states is well possible.

## 5 Discussion

Acoustic cavitation is a multi-parameter phenomenon, and it is well-known to be sensitive to liquid temperature and dissolved gas type and concentration, among several other factors like frequency and nuclei distribution or transducer and reactor geometry. In the present process of sequential or simultaneous application of US and MW irradiation, a particular interaction of temperature changes, gas saturation values, and ultrasonic degassing seems to play a role.

**Fig. 13:**
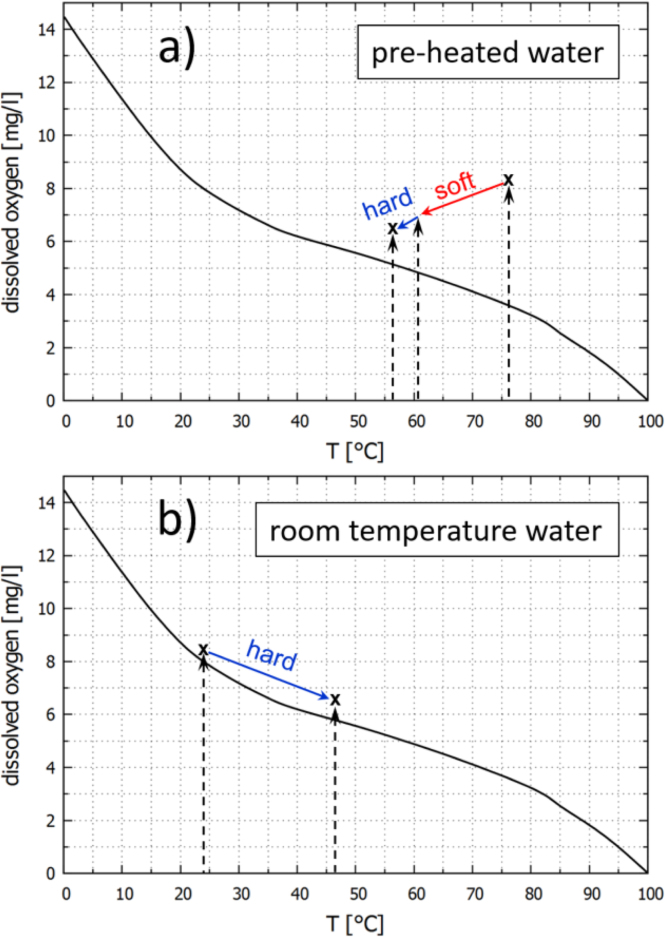
Progression of the experiments in the state space of temperature and dissolved oxygen concentration: (a) Pre-heated water, only US, Fig. 6. (b) Room temperature water, only US, Fig. 7. The black reference curve represents the equilibrium saturation of oxygen in water under air atmosphere at standard conditions, as obtained from [37]. Red arrows mark soft cavitation, blue arrows denote hard cavitation. Initial and end points (crosses) and transition temperatures (positions of vertical dashed arrows) were measured, intermediate progression is conjectured.

**Fig. 14:**
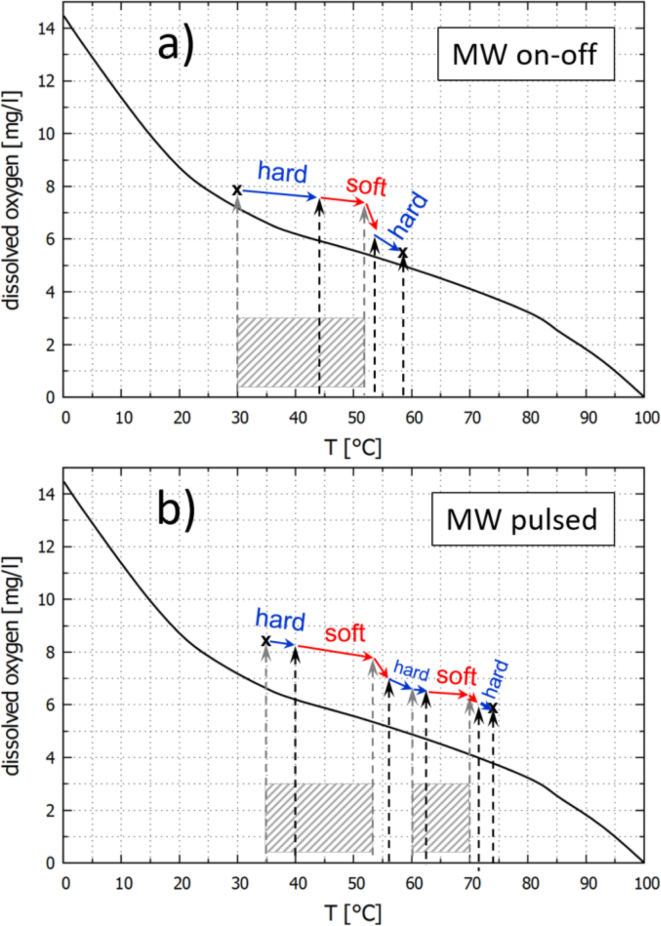
Progression of the experiments in the state space of temperature and dissolved oxygen concentration: (a) Room temperature water, US + MW on-off, Fig. 8. (b) Slightly pre-heated water, US + MW pulsed 2 min on–off, Fig. 10. The black reference curve represents the equilibrium saturation of oxygen in water under air atmosphere at standard conditions, as obtained from [37]. Red arrows mark soft cavitation, blue arrows denote hard cavitation. Initial and end points (crosses) and transition temperatures (positions of vertical dashed arrows) were measured, intermediate progression is conjectured. The gray shaded regions indicate the phases of MW on.

To discuss the results of the presented experiments, the plane of dissolved oxygen concentration (proportional to dissolved air concentration) vs. water temperature is depicted in Fig. 13, Fig. 14 for some of the shown experiments. Included is the curve of equilibrium saturation under standard atmospheric conditions (black). Generally, the equilibrium saturation concentration falls monotonously with temperature, which leads to the water being oversaturated if heated without degassing. This will be the case if the heating happens sufficiently rapid and without agitation. On the other hand, it is well-known that acoustic cavitation has a certain degassing effect by mechanical and diffusional mechanisms [25], [38], [39]. Therefore, intense sonication will counteract the tendency to oversaturate via heating to a certain extent.

The case shown in Fig. 13(a) represents the scenario from Fig. 6. There, sonication started in pre-heated, well oversaturated liquid and immediately resulted in soft cavitation. In the course of the shown experiment, the system finally reached the hard cavitation regime via cooling and degassing.

The second case in Fig. 13(b) corresponds to the experimental data from Fig. 7. With the liquid at room temperature and with dissolved air close to equilibrium, the system started already in a hard cavitating regime. During the run, the tendency for oversaturation due to the temperature rise was fully compensated by ultrasonic degassing: The system was able to run along the equilibrium line and maintain a state of inertial cavitation.

In the third case Fig. 14(a) that corresponds to the MW on–off data from Fig. 8, however, the heating by simultaneous MW irradiation is triggering soft cavitation after some time. The experiment from Fig. 10 is depicted as well and occurs in the phase diagram Fig. 14(b). The same scenario appears in a repeated fashion there: During both MW episodes, soft cavitation sets in, returning to the hard regime only after switch off.

Potential explanations for this observation could be one of the following:

(i) Due to the high rate of heating of the MW, the counteracting degassing via inertial cavitation cannot keep up with oversaturation, and it switches to a soft cavitation regime. Some time after switch-off of the microwave, hard cavitation returns and keeps the system close to the saturation curve, since its degassing potential can again compensate the oversaturation occurring at lower heating rate (induced solely by US). Currently we favor this reasoning, and conjectured paths of the systems are included in Fig. 14(a) and (b) by the blue and red arrows.

(ii) MW heating might happen rather inhomogeneous (compare for instance [22]), and some MW hot spot is locally heating up the water extremely. This could lead to very strong oversaturation and release of a large amount of dissolved gas and possibly vapor, which in turn triggers a transition to gassy cavitation. The relatively long delay between MW initiation and transition of cavitation regime, however, would weaken this argument.

(iii) Cavitation bubbles might contribute to inhomogeneous heating, but in a “microscopic” way: The MW absorption of liquid water in the presence of gas, vapor and phase boundaries potentially changes, which might cause a stronger, local heat-up around bubbles. This is a rather hypothetical point yet.

(iv) Further, and even more hypothetical, the degassing phenomenon might be influenced on the molecular scale, i.e., the MW fosters locally the gas concentration and thus amplifies outgassing (apart from the pure thermal effect by lowering the saturation level).

To clarify the reason behind the MW “softening” of cavitation, experiments with more frequent gas concentration monitoring might be conducted, as well as variation of the heating rates by US and MW and/or the treated liquid volumes. Our experiments indicate (effective) heating rates of about 2–3 deg/min as sustainable by the inertial cavitation regime (US of about 30 W applied to 100 ml of water), but the higher rates of about 5–7 deg/min (US ∼20 W in combination with MW 30 W) to 10 deg/min (US ∼20 W with MW 80 W) were transferring the system into gassy cavitation within about 30–60 s. Such values, however, should be taken with care for prediction of other setups, since several further aspects might play a role (e.g. the geometries of transducer, vessel and microwave application, acoustic frequency and intensity). Additional experiments might check the role of water vapor. By working with pre-heated and strongly degassed water samples, one might work at high temperature but avoid supersaturation.

As suggestions for optimizing combined US/MW setups, one might first clarify if soft cavitation would be detrimental or possibly even beneficial for the process in mind. Different driving schemes could result, e.g. pulsing MW in a faster frame, trying to avoid the delayed transition to gassy states. Also, variations of US and MW power, cooled vessels or degassing protocols before processing might help in reducing the risk of soft cavitation. In any case, a microphone monitor of combined or alternating US/MW setups can be recommended as an additional, rather simple process control. Probably a more thorough analysis of the airborne sound spectra than done in this work can give even more detailed information on the actual cavitation states in such systems.

From the phase diagrams in Fig. 13, Fig. 14, one might be tempted to extract something like the transition line between soft and hard cavitation, located somehow above the equilibrium saturation line. This could indeed be accomplished by preparation of many experiments similar to that in Fig. 6, but with a variation of initial temperatures and with a method to obtain as well oxygen levels at the transition. However, most probably the transition points depend on several further parameters of the setup, e.g., power settings, geometrical factors, and possibly even the liquid quality or history. Finally, a certain inherent randomness is to be expected as well, since cavitation multi-bubble fields are involved. Thus, exploration of a hard-soft cavitation diagram in the temperature-gas saturation parameter plane might be worth doing for a specific system or application, but probably it does not have a universal shape, and extrapolation to other systems might not be possible.

One might further ask if the energy conversion of MW and/or US to heat is affected by the cavitation regime. In the experiments shown, the slope of the temperature curve corresponds to the momentary heating power delivered to the liquid, and a change of the slope at a point of switching regimes could indicate exactly such an effect: the MW or US energy is better (rising slope) or worse (reduced slope) converted to heat in dependence of hard or soft cavitation. Indeed, one can observe such bends of the temperature curve at several switching points in the diagrams. For instance, in Fig. 8, Fig. 9, the slope is changing in an upward way when hard cavitation sets in again (at t≈5  min and t≈9  min), which would indicate better US energy conversion under hard cavitation. However, although such bends of the T curve apparently exist in the data, they are not always conclusive. An own dedicated study might give here more insight in the future.

## 6 Conclusions

The combined application of ultrasound of 20.5 kHz and microwave radiation on water has been investigated in terms of the acoustic emissions and cavitation regimes. Additional experiments with sonication of pre-heated and room temperature samples without MW helped in relating high-speed observations of cavitation bubbles with hydrophone and air microphone spectra. Essentially, two regimes of cavitation have been observed, that could be distinguished by the sound signals: a soft (gassy) cavitation state where large and weakly collapsing bubbles occur with acoustic emissions remaining on a lower level, and a hard (inertial) cavitation regime with smaller and stronger collapsing bubbles where acoustic emissions and broadband noise are on a high level. As a particular finding, turning MW on and off during sonication can alternately switch – with some time delay – between soft (MW on) and hard (MW off) cavitation regimes. This effect might be attributed to the high heating rate applied via the MW, rather than the absolute temperature of the water, since the emerging relative supersaturation with air cannot be equilibrated. While ultrasonic cavitation in the inertial regime produces apparently sufficiently degassing during slower heating to remain near equilibrium saturation, a faster heat deposition can lead to a switching towards a gassy cavitation state. This could compromise sonochemical applications with need for inertial cavitation and hot spots inside the bubbles, but might as well have beneficial effects in other processes, since synergetic effects have been observed for simultaneous US/MW irradiation [23]. One positive point might be a faster degassing of the liquid under MW assist.

From the presented results, further experimental investigations could be derived, together with potential strategies to optimize combined US/MW setups. Acoustic monitoring via analysis of airborne sound emissions is suggested to help in this respect.

## CRediT authorship contribution statement

**Dwayne Savio Stephens:** Writing – original draft, Software, Methodology, Investigation, Formal analysis, Conceptualization. **Adriano Troia:** Resources, Investigation. **Giancarlo Cravotto:** Writing – review & editing, Supervision, Conceptualization. **Katia Martina:** Writing – review & editing, Writing – original draft, Supervision, Investigation, Conceptualization. **Robert Mettin:** Writing – review & editing, Writing – original draft, Supervision, Project administration, Funding acquisition, Formal analysis, Data curation, Conceptualization.

## Declaration of competing interest

The authors declare that they have no known competing financial interests or personal relationships that could have appeared to influence the work reported in this paper.

## Figures and Tables

**Fig. A.15:**
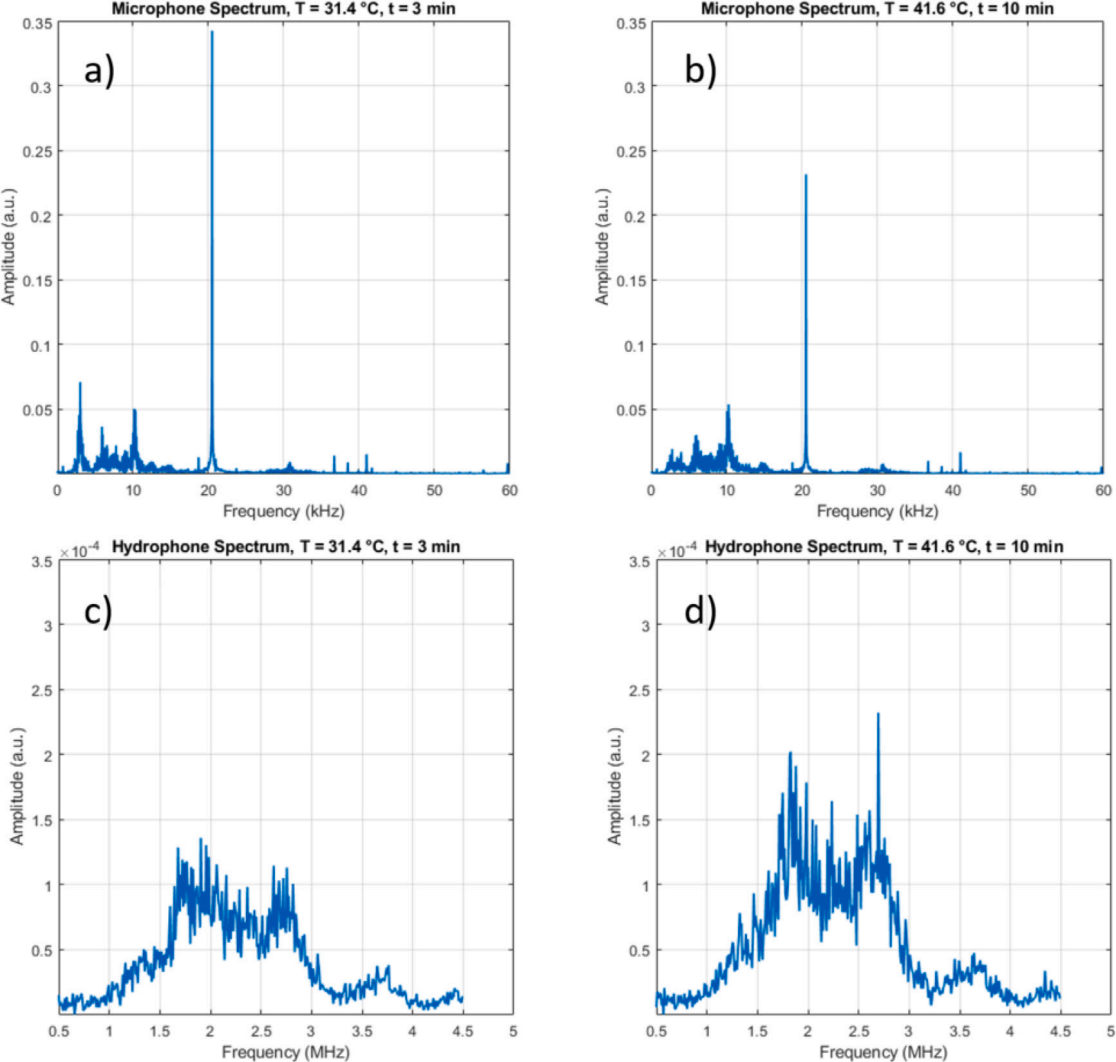
Sample spectra of two time instants marked in Fig. 7, room temperature water sonicated continuously. The actual temperature and sample time of the spectral recordings is denoted above the plots. (a) and (b) show microphone spectra up to 60 kHz, both indicating an inertial cavitation regime by a rather high noise floor around and below 10 kHz. Simultaneously measured hydrophone spectra from 0.5 to 5 MHz are shown in (c) and (d). They contain as well a significant broadband noise level.

**Fig. A.16:**
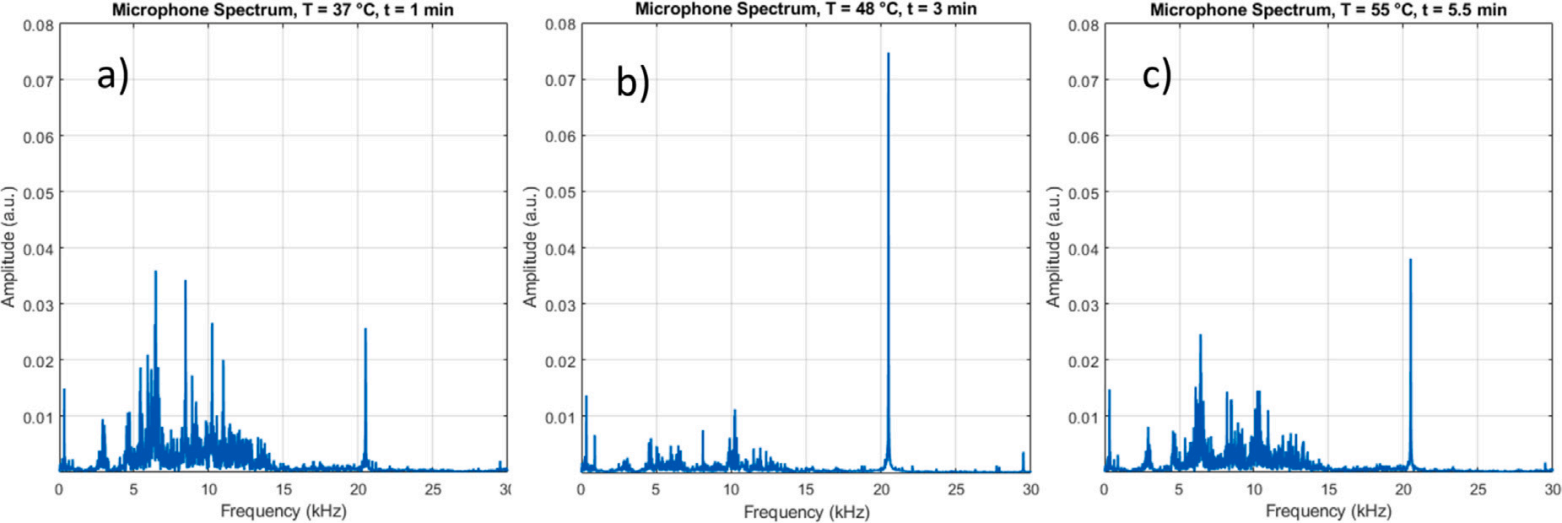
Sample microphone spectra (up to 30 kHz) of three time instants marked in Fig. 8, room temperature water continuously sonicated during and after a MW on-off switching. The actual temperature and sample time of the spectral recordings is denoted above the plots. Initially, a high noise level around and below 10 kHz exists, indicating a state of hard cavitation (a). The reduced noise level and an increased fundamental is visible in the soft cavitation regime during the MW application (b). After switching off the microwave, the noise level nearly returns to the previous height (c), indicating a return to hard cavitation.

**Fig. A.17:**
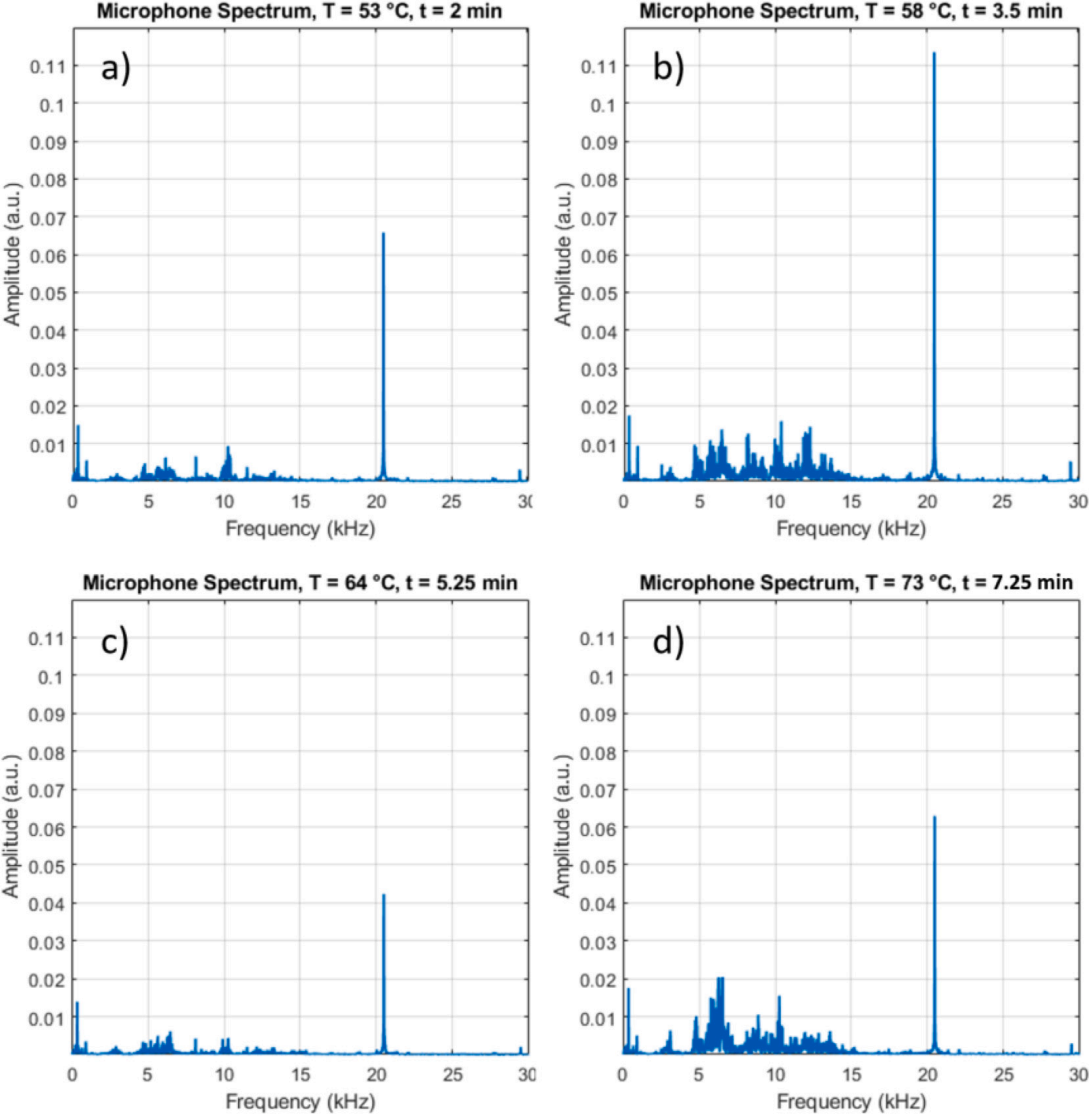
Sample microphone spectra (up to 30 MHz) of four time instants marked in Fig. 10, room temperature water continuously sonicated during and pulsed MW application. The actual temperature and sample time of the spectral recordings is denoted above the plots. The system switches (with some delay) in parallel with MW application from soft (a) for MW on to hard (b) cavitation for MW off. This happens repeatable, see (c) and (d). The hard cavitation state shows again a significant increase of the noise level in the subharmonic range; the fundamental rises here as well.

**Fig. B.18:**
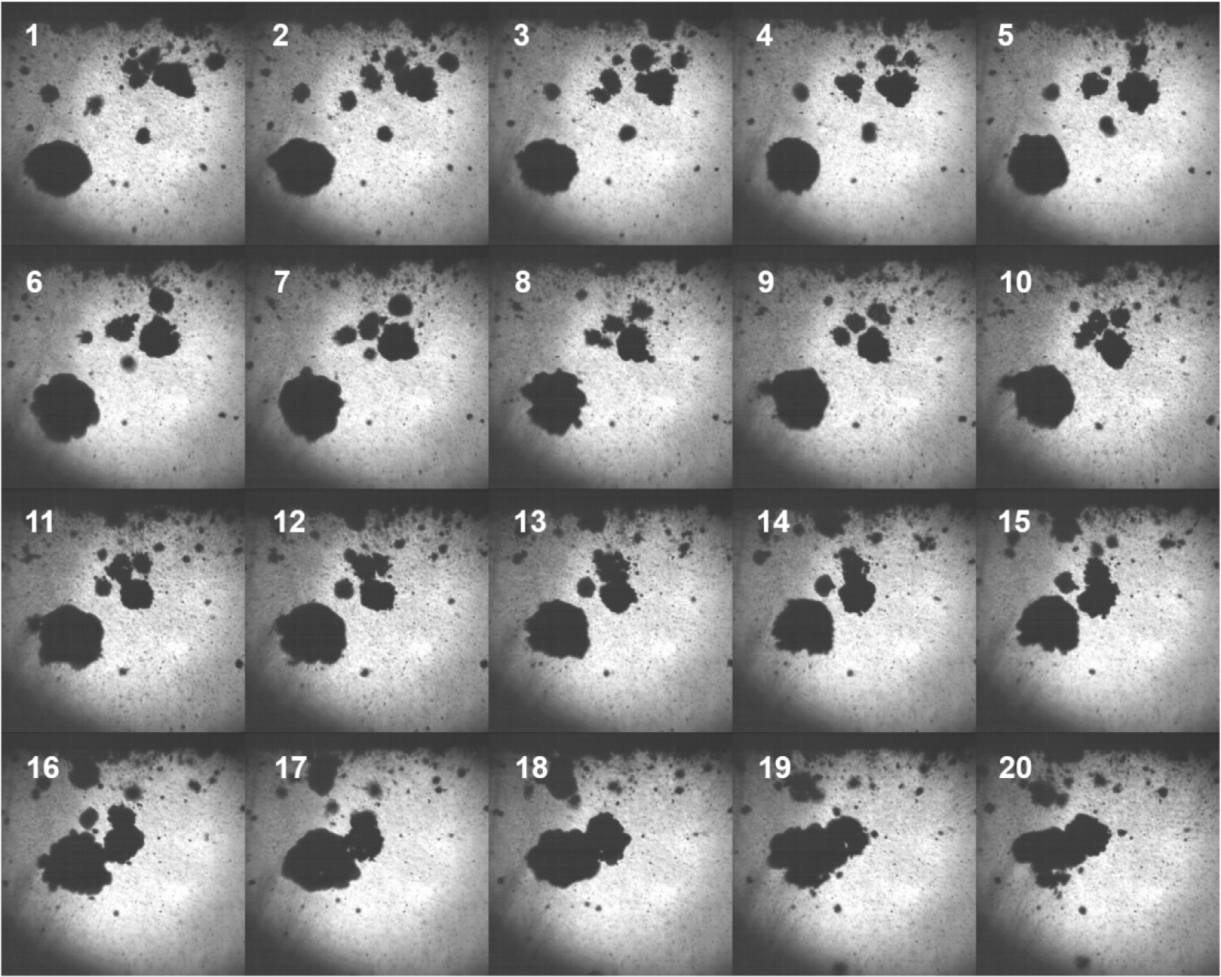
Frames from a high-speed recording series of soft cavitation in setup II. Frame width 6 mm, interframe time 0.2 ms, exposure time 20μs. The movie can be found in the Supplementary Material ((Link to the movie “SoftCavitation.avi”)).

**Fig. B.19:**
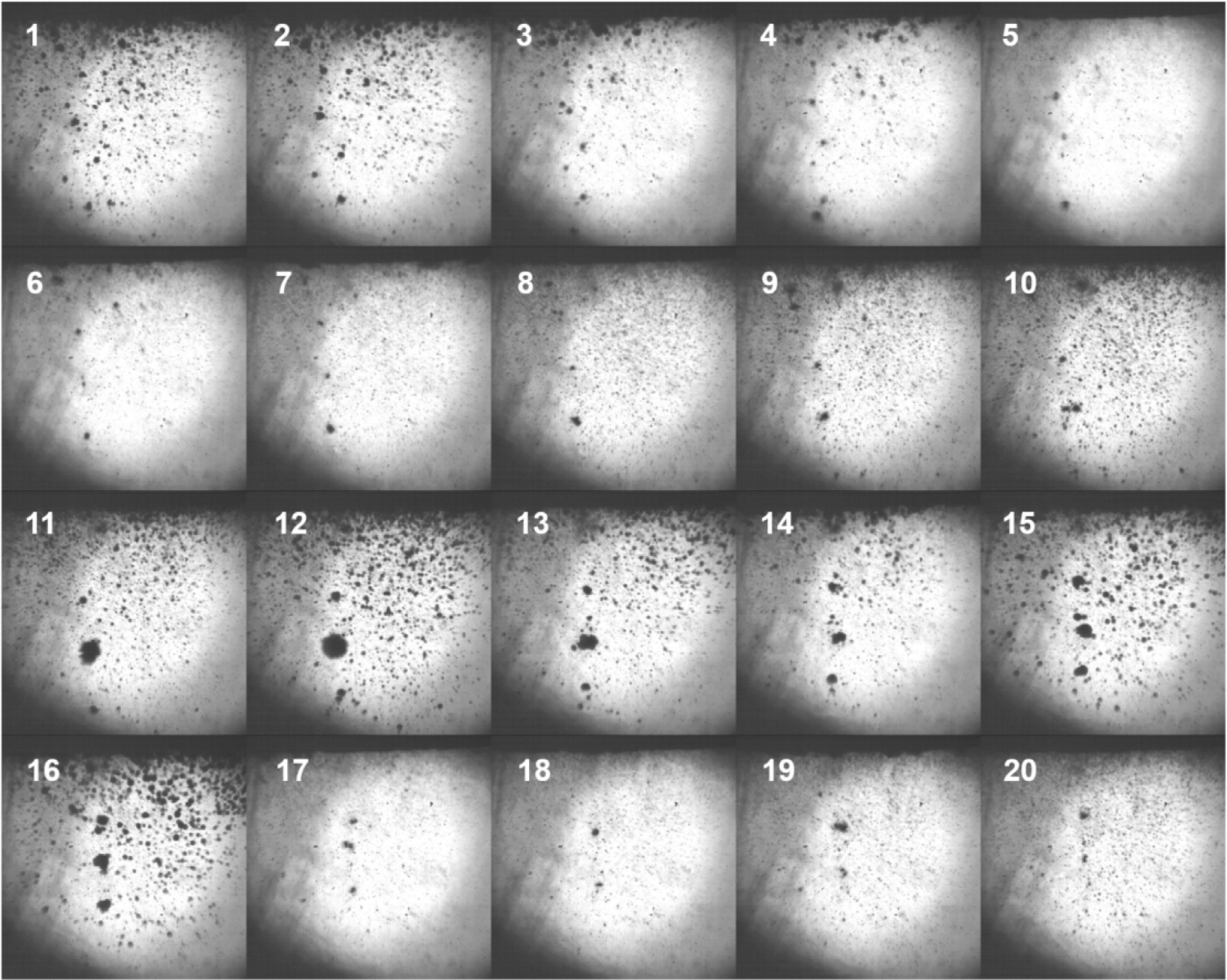
Frames from a high-speed recording series of hard cavitation in setup II. Frame width 6 mm, interframe time 0.2 ms, exposure time 20μs. The movie can be found in the Supplementary Material ((Link to the movie “HardCavitation.avi”)).

**Fig. B.21:**
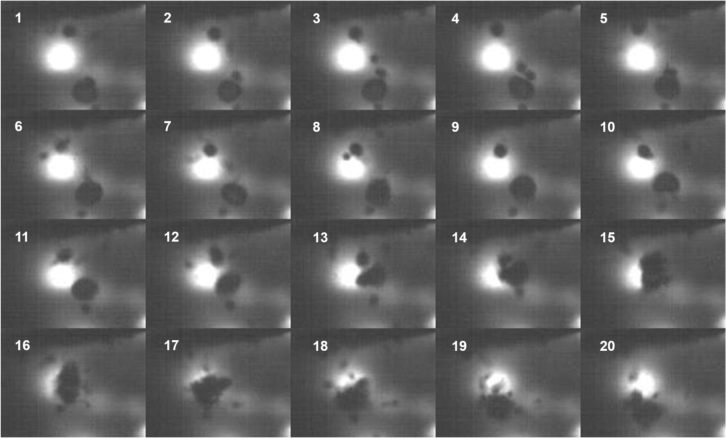
Frames from a high-speed recording series of soft cavitation in setup III. Frame width 8.2 mm, interframe time 1 ms, exposure time 1 ms. ((Link to the movie “SoftCavitation_in_MW.avi”)).

## Appendix A Sample acoustic spectra

Here we show for illustration some raw amplitude spectra of microphone and hydrophone recordings of the experiments discussed in Section 4, see Fig. A.15, Fig. A.16, Fig. A.17. They represent soft and hard cavitation regimes, and the exact positions are marked in the respective time trace plots.

## Appendix B High-speed recordings

For illustration, we show a collection of high-speed frames of typical recordings of soft and hard cavitation. The first two videos, Fig. B.18, Fig. B.19, were recorded in an experiment with pre-heated water in setup II, (compare Fig. 5, Fig. 6). Since the framerate was 5000 fps and the US frequency was f=20500  Hz at 45 W, the interframe time of 0.2 ms corresponds to 4.1 driving periods T=1/f of the US field. Due to the short exposure time of 20μs, the bubbles appear more or less sharp. Each frame leaves out 4T and then shifts the relative phase between driving and camera exposure by 10% of T. Thus a collection of 20 subsequent frames gives the apparent impression of bubble dynamics during 2T, virtually sampled with 205 kfps.

The sequence in Fig. B.18 was taken immediately after start of sonication. Large bubbles with surface instabilities occur, but no clear phase of bubble collapse can be discerned. This is an indication of the soft, gaseous cavitation state. The sequence in Fig. B.19 was recorded after 4.5 min with US on. Now, large bubbles are gone and many smaller bubble populate the field. A clear pulsation of the bubble field can be identified, i.e., on some frames nearly all bubbles (dark spots) seem to disappear since they are collapsed to very small size. The strong volume oscillations of the bubbles indicate a hard, inertial cavitation regime. Both movies are provided in the Supplementary Material.

Recordings from within the MW oven are shown in Fig. B.20, Fig. B.21. Here, the experiment was run in setup III under 100 W microwave heating of room temperature water and US at 30 W, similar (but not identical) to Fig. 8. Frame rate was only 1000 fps at an extended exposure time of 1 ms, which blurred the image significantly. The acoustically judged transition from hard to soft cavitation occurred at 35 °C, and the sequence of Fig. B.20 had been taken just before this happened. Small bubbles and fast changing bubble streamer structures are discernible. Fig. B.21 shows frames of a recording directly after the transition. Now, larger and slower moving bubble conglomerates are visible. Taking the lower spatial and temporal resolution and poor image quality into account, an optical confirmation of the different cavitation regimes inside the MW oven can fairly be stated. Both movies are also contained in the Supplementary Material.

## Appendix C Supplementary data

Video S1 Four movies showing soft and hard cavitation outside and inside a microwave oven.
